# The Subcellular Localization of the Receptor for Platelet-Activating Factor in Neutrophils Affects Signaling and Activation Characteristics

**DOI:** 10.1155/2013/456407

**Published:** 2013-08-29

**Authors:** Emil Andréasson, Karin Önnheim, Huamei Forsman

**Affiliations:** Department of Rheumatology and Inflammation Research, Göteborg University, Guldhedsgatan 10A, 413 46 Göteborg, Sweden

## Abstract

The localization in neutrophils, of the receptor for platelet-activating factor (PAFR), has been determined using subcellular fractionation and a receptor mobilization protocol. We show that the PAFR is expressed primarily in the plasma membrane. Although activation of neutrophils by PAF induces responses typical also of agonists that bind the formyl peptide receptors (FPR), known to be stored in mobilizable organelles, some quantitative as well as qualitative differences were observed when neutrophils were activated through these receptors. PAF is equipotent to fMLF (high affinity agonist for FPR1) to cleave off L-selectin and to induce granule/vesicle secretion but is more potent than fMLF to induce a rise in intracellular Ca^2+^. Similar to fMLF, PAF induced also a robust release of reactive oxygen species, but with higher EC_50_ value and was less sensitive to a PI3K inhibitor compared to the fMLF response. Despite the lack of a granule localized storage pool of receptors, the PAF-induced superoxide production could be primed; receptor mobilization was, thus, not required for priming of the PAF response. The desensitized PAFR could not be reactivated, suggesting that distinct signaling pathways are utilized for termination of the responses triggered through FPR1 and PAFR.

## 1. Introduction

Neutrophil granulocytes, professional phagocytes of the innate immune system, are recognized and activated by chemoattractants, soluble molecules serving as “danger signals” [1]. Activation of the phagocytes is of great importance for the outcome of the continuously ongoing combat with invading microorganisms, but accumulation of these cells and their subsequent release of reactive oxygen species (ROS) and proteolytic enzymes are also responsible for the tissue damage associated with a number of inflammatory disease conditions [2]. Research on the structure/functional relationship of neutrophil chemoattractants and their receptors as well as the downstream signaling pathways is therefore of direct clinical importance and relevance, and the list of structurally well-characterized receptor agonists as well as antagonists/inhibitors has steadily grown [3, 4]. 

All chemoattractant receptors including the most extensively studied formyl peptide receptors (FPRs) exhibit some sequence homologies and belong to the family of pertussis toxin sensitive G protein-coupled family of receptors (GPCRs). The neutrophil GPCRs specifically recognize different agonists that most commonly are naturally occurring peptides/proteins with a defined structure. There are, however, also some receptors that display high affinity binding for lipid metabolites such as lipoxin A4 (LXA4), leukotriene B4 (LTB4), and platelet-activating factor (PAF) [5–7]. The latter was the first bioactive phospholipid identified, and it has a defined and characteristic structure (an alkyl ether linkage at the *sn*-1-position of the glycerol backbone), and is named after its effect on platelets, activation and aggregation [8, 9]. This lipid agonist exerts all its basic functions through binding to and activation of the G-protein-coupled PAF receptor (PAFR) [3, 10]. Research during the last years has demonstrated important roles for PAF in many pathophysiological conditions including asthma, psoriasis, and endotoxic shock [11]. Additionally, PAFR is expressed in neutrophils, and PAF activated neutrophils also serve a prominent source for the generation of PAF and other lipid inflammatory mediators such as LTB4 suggesting a role for this receptor/ligand pair not only in host defence but also in modulating inflammatory responses. Similar to most neutrophil GPCRs, occupation of the PAFR promotes secretion of granule constituents and mobilization of cell surface receptors, cellular migration [12], and priming of the cells to generate increasing amounts of ROS [13]. PAF has been shown by others to directly activate the electron transporting system (the NADPH oxidase) that generates ROS in eosinophils, and in our hands PAF also directly activates this system in neutrophils [14]. 

Some chemoattractants are rather poor ROS inducers in neutrophils, but the response induced can be dramatically increased (primed) by nonactivating agents such as tumor necrosis factor alpha (TNF-*α*) and lipopolysaccharides (LPS) derived from Gram-negative bacteria [15, 16]. The priming phenomenon has been extensively investigated, and its biological significance in many disease processes has been well documented. With respect to priming of neutrophils in response to endogenous lectins of the galectin family and receptor specific agonists for FPR1 and FPR2, we and others have suggested granule mobilization resulting in surface receptor upregulation as a key event [17, 18]. Accordingly, in resting neutrophils, the mobilizabled FPRs and receptors for galectins are found primarily in secretory organelles that fuse with the plasma membrane in response to different types of secretagogues [19–22]. The precise subcellular localization of PAFRs in human neutrophils has, however, not yet been examined.

In the present study, we determined the subcellular localization of the PAFR in resting neutrophils, and our studies demonstrate that PAFRs are localized in a light membrane fraction containing plasma membranes and secretory vesicles. Mobilization data show that the cells lack an easily mobilizable PAFR pool, suggesting that the receptor is present primarily in the plasma membrane. Still, the PAF-induced ROS production in neutrophils could be enhanced by TNF-*α* and cytoskeleton disrupting agents, suggesting that our proposed mechanism for priming does not apply for the PAF/PAFR receptor-ligand pair. The PAFR shared many signalling properties and basic functional characteristics with the FPRs, but there were also many quantitative as well as qualitative differences, possibly linked to the difference in subcellular localization between the two receptors. 

## 2. Materials and Methods

### 2.1. Chemicals

The hexapeptide WKYMVM was synthesized, and HPLC purified by KJ Ross-Petersen (Holte, Denmark). The formylated tripeptide formyl-methionyl-leucyl-phenylalanine (fMLF), isoluminol, cytochalasin B (CytB), pertussis toxin (PTX), TNF*α*, latrunculin A, and phorbol myristate acetate (PMA) were obtained from Sigma (Sigma Chemical Co., St. Louis, MO, USA). Peptides were dissolved in DMSO and stored at −70°C until use. Subsequent dilutions of all peptides were made in Krebs-Ringer phosphate buffer (KRG, pH 7.3; 120 mM NaCl, 5 mM KCl, 1.7 mM KH_2_PO_4_, 8.3 mM NaH_2_PO_4_, and 10 mM glucose) supplemented with Ca^2+^ (1 mM) and Mg^2+^ (1.5 mM). The PAFR antagonist WEB2086 was from Tocris bioscience (Bristol, UK), and PAF was from Avanti Polar Lipids (Avanti Polar Lipids. Inc, Alabama). Ficoll-Paque was obtained from Amersham Pharmacia Biotech AB (Uppsala, Sweden). Horseradish peroxidase (HRP) was obtained from Boehringer Mannheim (Germany). Kinase inhibitors were obrained from Calbiochem (Darmstadt, Germany). FURA-2 was from Molecular Probes (Eugene, OR, USA). The MMP9 ELISA was from R&D systems, the ELISA-kit for NGAL was from Bioporto Diagnostics. Antibodies against CR3, and L-selectin were from BD Biosciences (Franklin Lakes, NJ, USA).

### 2.2. Isolation of Human Neutrophils

Human peripheral blood neutrophils were isolated from buffy coats from healthy blood donors using dextran sedimentation and Ficoll-Paque gradient centrifugation as described [23]. The remaining erythrocytes were hypotonic lysed, and the neutrophils were washed twice and resuspended in KRG, and stored on melting ice until use. This isolation process permits cells to be purified with minimal granule mobilization. 

### 2.3. Neutrophil NADPH-Oxidase Activity

The NADPH-oxidase activity was determined using isoluminol-enhanced chemiluminescence (CL) [24, 25]. The CL activity was measured in a six-channel Biolumat LB 9505 (Berthold Co., Wildbad, Germany), using disposable 4 mL polypropylene tubes with a 900-microliter reaction mixture containing 10^5^ cells, isoluminol (2 × 10^−5 ^M), and HRP (2U). Intracellular ROS was measured by luminol (cell permeable) in the presence of superoxide dismutase and catalase [25]. The tubes were equilibrated in the Biolumat for 5 min at 37°C, after which the stimulus (100 *μ*L) was added, and the light emission was recorded continuously. By a direct comparison of the superoxide dismutase-inhibitable reduction of cytochrome c and superoxide dismutase-inhibitable CL, 7.2 × 10^7^ counts were found to correspond to the production of 1 nmol superoxide (using a millimolar extinction coefficient for cytochrome c of 21.1). When experiments were performed with priming and antagonists/inhibitors, these substances were included in the CL reaction mixture for different time period (control cells received no treatment but were incubated at the same condition) before stimulation with agonists. 

### 2.4. Subcellular Fractionation and Marker Analysis

Subcellular fractionation was performed as described [26]. Briefly, neutrophils were treated with the serine protease inhibitor, diisopropyl fluorophosphates (DFP, 8 *μ*M), disintegrated by nitrogen cavitation (Parr Instruments Co., Moline, IL), and the postnuclear supernatant was fractionated on two- (1.05 and 1.12 g/L) or three-layer (1.12, 1.09 and 1.05 g/L) Percoll gradients and centrifuged at 15,000 g for 45 minutes in a fixed-angle JA-20 Beckman rotor. Fractions of 1.5 mL were collected by aspiration from the bottom of the centrifuge tube. The localization of subcellular granules was determined by granule marker analysis. Alkaline phosphatase (marker for secretory vesicles/plasma membrane) was measured by hydrolysis of p-nitrophenyl phosphate, at pH 10.5, in a sodium barbital buffer. Myeloperoxidase (a marker for azurophil granules), and gelatinase, and neutrophil gelatinase-associated lipocalin (NGAL) were measured by Western blotting and ELISA, respectively. 

### 2.5. SDS-PAGE and Western Blotting

Percoll gradient fractions, prepared as describe above, were diluted in nonreducing sample buffer, boiled for 5 min, and applied to 12% SDS-polyacrylamide gels. The separated proteins were transferred to nitrocellulose membranes, followed by blocking the membrane at room temperature for 1 hr in 1% BSA. After blocking, the blots were incubated overnight at 4°C with primary antibody against PAFR (Cat number SC-8742, Santa Cruz biotechnology; an affinity purified polyclonal goat antibody directed against a peptide at the C-terminus of the human receptor). Nonbound antibodies were removed by washing with PBS Tween. An HRP-conjugated secondary antibody was used for visualization of the receptors. 

### 2.6. Calcium Mobilization

Cells at the density of 1–3 × 10^6^ per mL were washed with Ca^2+^-free KRG and centrifuged at 220 ×g. The cell pellets were resuspended at a density of 2 × 10^7^ cells/mL in KRG that contained 0.1% BSA and loaded with 2 *μ*M Fura 2 AM for 30 minutes at room temperature. The cells were then diluted to twice the original volume with RPMI 1640 culture medium without phenol red (PAA Laboratories GmbH, Pasching, Austria) and centrifuged. Finally, the cells were washed once with KRG and resuspended in the same buffer at a density of 2 × 10^7^ cells/mL. Calcium measurements were carried out in a Perkin Elmer fluorescence spectrophotometer (LC50), with excitation wavelengths of 340 nm and 380 nm, an emission wavelength of 509 nm, and slit widths of 5 nm and 10 nm, respectively. The transient rise in intracellular calcium is presented as the ratio of fluorescence intensities (340 nm : 380 nm) detected. The measuring cuvette contained catalase (2000 U) to counteract inactivation of the chemoattractants by the MPO-H_2_O_2_-system [27].

The concentration of EGTA required to achieve a calcium-free environment was determined by titration of the ionomycin-triggered production of oxidants by neutrophils as described [27]. A small volume (10 *μ*L) of EGTA-containing buffer was added to the measuring vial, and 20 seconds later the cells were activated by the addition of ionomycin (5 × 10^−7^ M final concentration). The lowest concentration of EGTA that inhibited the ionomycin-induced response maximally (around 90%) was used in subsequent studies to ensure that no Ca^2+^ entered the cells across the plasma membrane. 

### 2.7. Granule Secretion and Assessment of Surface Molecules by FACS and ELISA

Human neutrophils (2 × 10^6^ cells) were stimulated with PAF, fMLF (100, 10 or 2 nM), or buffer as control and incubated at 37°C for 10 min. Samples were placed on ice and centrifuged at 335 ×g for 10 min at 4°C. Supernatants were removed and centrifugated once more at 1500 ×g for 5 min, then stored at −70°C, and used for marker analysis. The cells were resuspended in ice-cold PBS, and 5 × 10^5^ cells/sample were labeled with antibodies against CR3 or L-selctin for 30 min at 4°C in the dark. The cells were then washed twice before resuspended in PBS and analysed by FACS. 

### 2.8. Statistic-Analysis

One-way Anova with Dunnett's multiple comparison test was used for statistical analysis. *P* < 0.05 was considered statistically significant.

## 3. Results

### 3.1. The PAFR Is Localized Primarily in the Neutrophil Plasma Membrane

**Figure 1:**
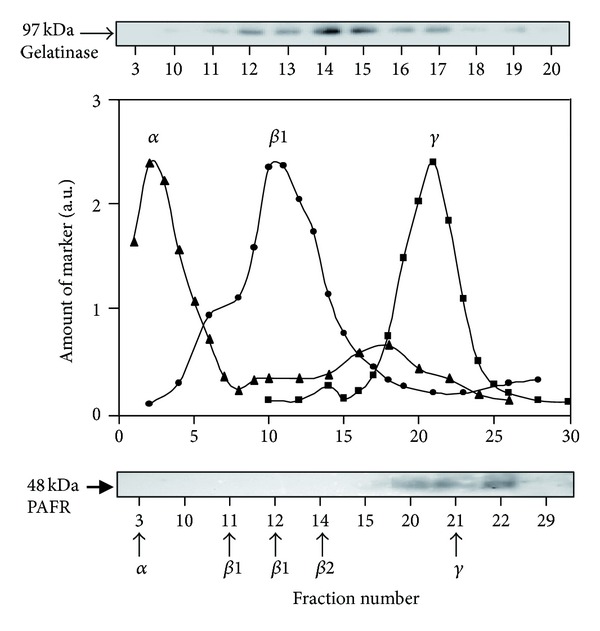
Subcellular localization of the PAFR in resting neutrophils. Neutrophil subcellular organelles from disintegrated cells were fractionated on a three-layer Percoll gradient. Upper panel. The distribution of gelatinase (marker for the *β*-fractions) visualized through immunoblotting. Middle panel: localization of azurophil granules (*α*-fraction; marker MPO) specific granules (*β*1-fraction; markers NGAL) and plasma membrane/secretory vesicles (*γ*-fraction; ALP). The markers (MPO = ▲; NGAL = ●; ALP = ■) are expressed in arbitrary units. Lower panel: proteins from selected fractions were separated by SDS-PAGE, and the localization of PAFR was determined by immunoblotting with a specific antibody against PAFR.

Only smaller fractions of earlier characterized neutrophil chemoattractant receptors such as the FPRs, belonging to the family of GPCRs, are localized in the plasma membrane, whereas most of these receptors are stored in the secretory organelles, that is, secretory vesicles and specific granules [18, 28, 29]. We have now determined the subcellular localization of the neutrophil receptor for PAF using a subcellular fractionation technique and a receptor mobilization protocol combined with FACS analysis. Two- or three-layer Percoll gradients were used, and the localization of PAFR was determined by immunoblotting with a receptor specific antibody. When analyzing the localization in a three-layer Percoll gradient, which can separate not only the main organelles, azurophil granules, and specific granules, from the light membrane fraction, but also the gelatinase granules from the somewhat denser specific granules (Figure 1 upper panel), it is clear that the PAFR cannot be found in the granules (Figure 1 lower panel). The PAFR was found only in the light membrane fraction enriched in plasma membranes and secretory vesicles (Figure 1 lower panel). This distribution was further confirmed using a two-layer Percoll gradient in which all the specific/gelatinase granules are concentrated in the same fractions (see supplementary Figure 1 in Supplementary Material available online at http://dx.doi.org/10.1155/2013/456407). 

**Figure 2:**
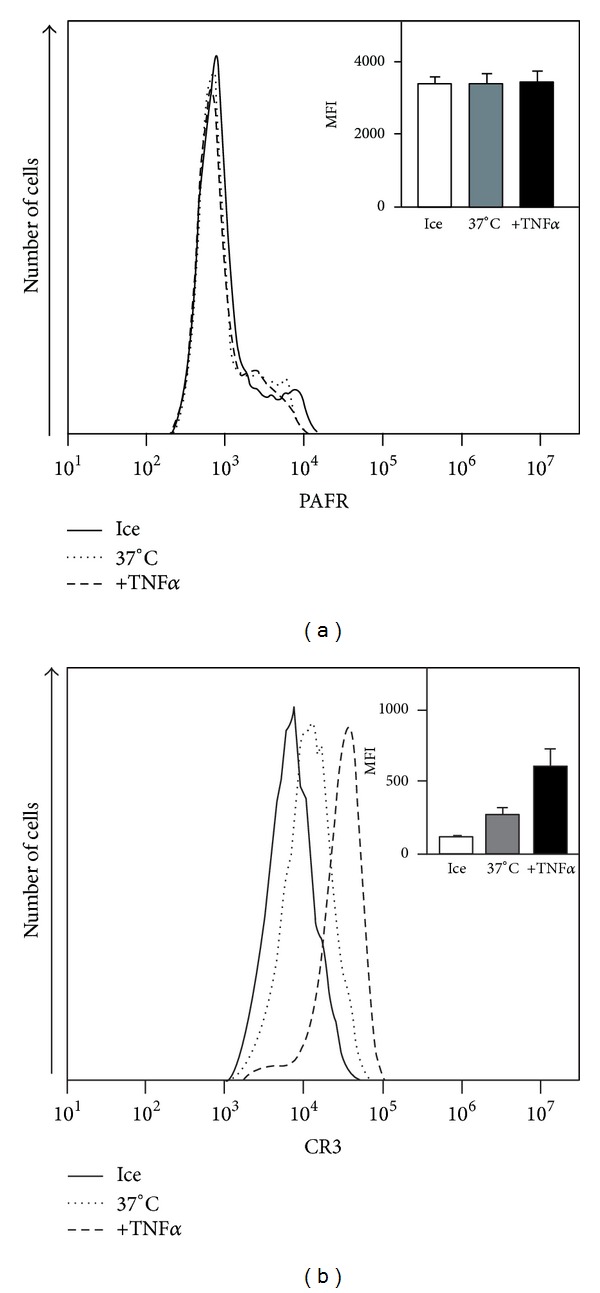
TNF-*α* induced receptor mobilization does not involve the PAFR. Human neutrophils (5 × 10^5^) were incubated on ice (solid lines), or at 37°C without additive (dotted lines), or with TNF-*α* (10 ng/mL; dashed lines) for 20 min. The cells were then fixed with 2% ice-cold paraformaldehyde and labeled with specific antibodies against PAFR (A) or complement receptor 3 (CR3; B). Surface receptor expression was determined by flow cytometry, and a representative histogram is shown. Abscissa: fluorescence intensity; Ordinate: number of cells. The insets show the mean fluorescence intensity (MFI) ± SEM (*n* = 5).

The secretory vesicles are very easy mobilized membrane storage organelles and upon secretion the receptors present in the vesicles are exposed on the cell surface [22, 30]. To determine the role of the easily mobilizable secretory organelles as a storage pool for PAFRs, neutrophils were incubated at 37°C for 20 min in the presence or absence of TNF-*α*. The cells gradually increased their surface expression of the marker control, CR3, a cell surface receptor localized not only in the plasma membrane but also in the secretory vesicles and in the secretory granules (Figure 2(b)). Mobilization of CR3 was not associated with any increase in the surface exposure of PAFRs indicating the lack of a mobilizable pool of PAFR in neutrophils (Figure 2(a)). Taken together, these data show that the PAFR is present primarily (or maybe even exclusively) in the plasma membrane. 

### 3.2. PAF Triggers Granule Secretion and a Release of ROS from Human Neutrophils

Interaction of neutrophils with PAF has been shown to induce many cellular responses including secretion of granule constituents. Accordingly, we show that the addition of PAF to human neutrophils activated the cells and induced granule secretion as reflected by a shedding of the adhesion molecule L-selectin (CD62L; a protein highly expressed on the surface of resting cells), an increased cell surface expression of CR3 (a marker for the secretory granules) and secretion of the granule localized proteins NGAL (a marker for specific granules) and gelatinase (a marker for gelatinase granules) (supplementary Figure 2). PAF was found to be as potent as fMLF (high affinity agonist for FPR1) and WKYMVM (high affinity agonist for FPR2) to cleave off L-selectin and to induce fusion between the granules/vesicles and the plasma membrane (shown for fMLF and PAF in supplementary Figure 2).

**Figure 3:**
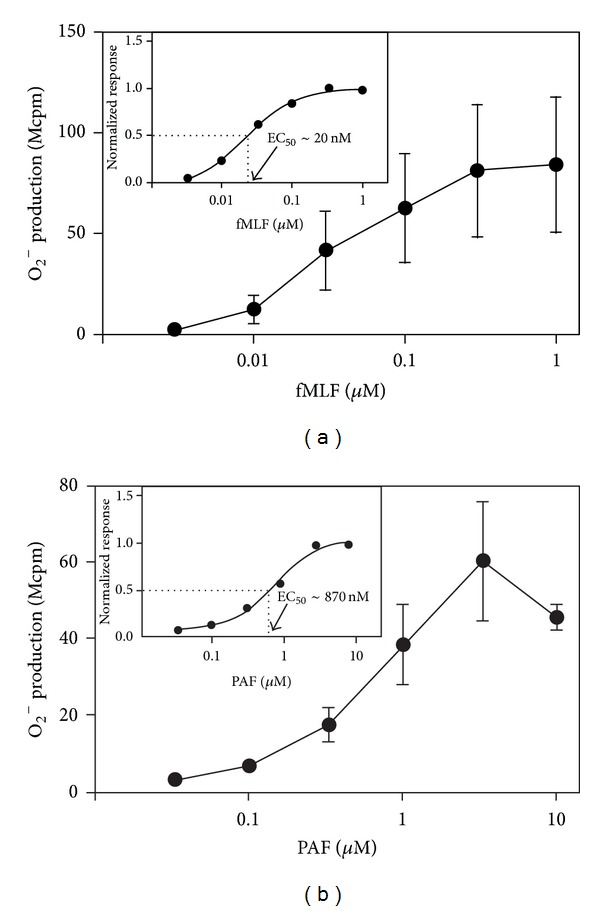
PAF and fMLF dose dependently trigger the release of superoxide anions from human neutrophils. Human neutrophils (10^5^ cells) were incubated at 37°C for 5 min in measuring vials containing isoluminol and HRP. Various concentrations of fMLF (A) or PAF (B) were added, and the release of superoxide anions was recorded continuously. Data are expressed as mean peak value ± SEM (*n* = 5). Abscissa, agonist concentration; ordinate, superoxide production expressed as counts per minute × 10^6^ (Mcpm). The insets show one representative normalized dose-response experiment with respective EC_50_ values calculated for fMLF (A) and PAF (B), respectively.

**Figure 4:**
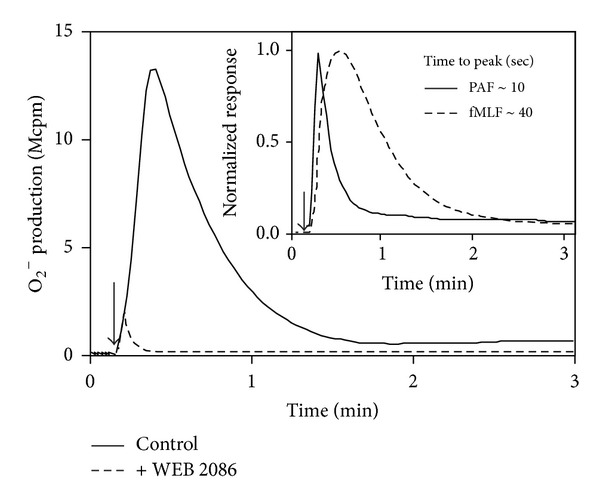
PAF triggers a rapid and WEB2086 sensitive release of superoxide anions from human neutrophils. Human neutrophils (10^5^ cells) were incubated at 37°C for 5 min in measuring vials containing isoluminol and HRP in the absence (control, solid line in the main figure) or presence of the PAFR antagonist WEB2086 (dashed line in the main figure). The cells were then activated with PAF (100 nM, added at arrow), and the release of superoxide anions was recorded continuously. Inset: the kinetics of superoxide production induced by PAF (100 nM; solid line) was compared to that induced by fMLF (100 nM; dashed line), and the times required to reach the respective peak of the response are given. Data are derived from one representative experiment out of at least five. Abscissa: time of study (min); ordinate, superoxide production expressed as counts per minute × 10^6^ (Mcpm; main figure) or normalized to compare the time courses (inset).

PAF is generally considered as a nonactivating or very poor/weak ROS inducer [13], contrasting the activity induced by many other chemoattractants such as fMLF which is regarded as a strong inducer. In our hands, PAF at a concentration of 100 nM promoted a robust release of ROS, and the magnitude of the response was comparable to that induced by fMLF and WKYMVM (shown for PAF and fMLF in Figure 3). The level of ROS production/release induced by PAF increased dose dependently with an EC_50_ of *≈* 800 nM (Figure 3(b)), which should be compared to an EC_50_ value of 50 nM for fMLF (Figure 3(a)). The time courses differed between the PAF- and the fMLF-induced cellular responses, in that the peak of the response was reached at an earlier time point, and the response declined to baseline somewhat more rapidly with PAF, compared to the FPR1-mediated or FPR2-mediated responses (shown for PAF and fMLF in Figure 4). No ROS production was detected intracellularly (data not shown) suggesting that PAF triggers exclusively an extracellular release of ROS in neutrophils. As expected, a specific PAFR antagonist (WEB2086) completely and selectively abolished the release of ROS upon PAF stimulation demonstrating that PAFR is the responsible receptor in mediating PAF-induced ROS production (Figure 4). Further, pertussis toxin abolished the PAF response showing that a pertussis-toxin sensitive G-protein is involved in signaling downstream of the PAFR (supplementary Figure 3). As a control, the cells treated with pertussis toxin were nonresponding also to fMLF but fully responsive to PMA (a ROS inducer that signals independent of the PTX-sensitive G-protein) (supplementary Figure 3).

Taken together, these data clearly show that PAF is not only a potent secretagogue but also a potent ROS activator, and the production is mainly due to an assembly of the oxidase in the plasma membrane and a release of the ROS. The PAF response reaches a peak value as well as returns to the baseline at an earlier time point than the responses mediated by the FPRs, suggesting that different signaling pathways are utilized downstream of the PAFR and the FPRs.

### 3.3. The PAFR Allows Priming but Not Reactivation

**Figure 5:**
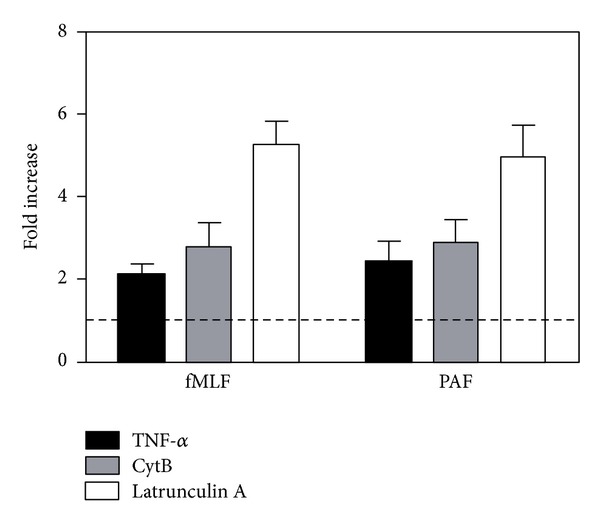
The PAF-induced neutrophil response is primed by TNF-*α* and inhibitors of actin polymerization. Human neutrophils were incubated at 37°C for 20 min with the priming agents TNF-*α* (10 ng/mL; black bars) or 5 min with either CytB (5 *μ*g/mL; grey bars) or latrunculin A (50 ng/mL; white bars). Control cells were incubated at the same conditions but in the absence of any priming agents. The cells were then activated with fMLF (100 nM) or PAF (100 nM), and the release of superoxide was recorded continuously. Data are expressed as fold increase of the peak values of the response from primed cells compared to nonprimed control (mean ± SEM; *n* = 3). The dashed line in the graph denotes the value expected with a nonactive priming agent.

Nonactivating concentrations of PAF primed neutrophils to enhanced ROS production with fMLF as the second agonist [31, 32], which is a well known phenomenon and it was to be expected since PAF is a potent secretagogue (supplementary Figure 2, [33]). Basically, priming is defined as a hyperreactive state of neutrophils induced through an exposure of the cells to a nonactivating priming agent such as TNF-*α*. To further elucidate the function of the PAFR, we determined whether PAF-mediated ROS production could be primed by well-known and earlier characterized priming agents, TNF-*α*, CytB, and latrunculin A. TNF-*α* treated neutrophils produced higher amount of ROS upon subsequent stimulation with PAF, compared to nontreated control cells (Figure 5), showing that also the PAF response could be primed. Additionally, we found that pretreatment of cells with the cytoskeleton disrupting agents CytB or latrunculin A significantly increased the amount of ROS release also in response to PAF (Figure 5). 

**Figure 6:**
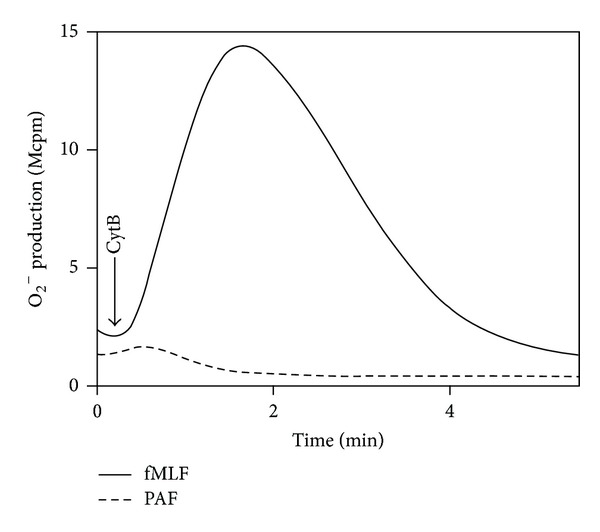
The desensitized PAFR is not reactivated by CytB. Neutrophil was desensitized with fMLF (100 nM; FPR1 agonist, solid line) or PAF (100 nM; PAFR agonist, dashed line) at 15°C for 10 min. The cells were then transferred to 37°C, and the incubation was continued for another 10 min. The cells were then challenged with CytB (5 *μ*g/mL, added at the time point indicated by arrow), and reactivation of the receptor was determined through the release of superoxide. One representative experiment out of three is shown. Abscissa: time of study (min); ordinate, superoxide production expessed as counts per minute × 10^6^ (Mcpm).

The signalling of an occupied GPCR rapidly ceases as the receptor is transferred to desensitized (nonresponding/signaling) state achieved in neutrophils through a binding of the ligand occupied receptor to the actin cytoskeleton [34–36]. Accordingly, when binding of the chemoattractant fMLF to FPR1 takes place at low temperature (≤15°C), the activating signalling state of the receptor is bypassed, and it is directly deactivated/desensitized, but the receptor can be reactivated/resensitized by cytoskeleton-disrupting drugs (Figure 6). Such cytoskeleton-dependent receptor reactivation occurs not only with desensitized FPR1, but also with FPR2 and C5aR (data not shown). Interestingly, preincubating cells with PAF at low temperature (≤15°C) deactivated the PAFR, but when transferred to 37°C these cells/receptors could not be reactivated by cytoskeleton disrupting agents (Figure 6). Taken together, our data show that PAF-induced ROS production can be enhanced by the priming agent TNF-*α* as well as through disruption of the cytoskeleton, but in contrast to FPR1, the desensitized PAFR could not be reactivated through a disruption of the cytoskeleton, suggesting that distinct signaling pathways are utilized for desensitization/termination of FPRs and PAFR. 

### 3.4. Basic Signaling Downstream of the PAFR Include a Release of Calcium from Intracellular Stores

**Figure 7:**
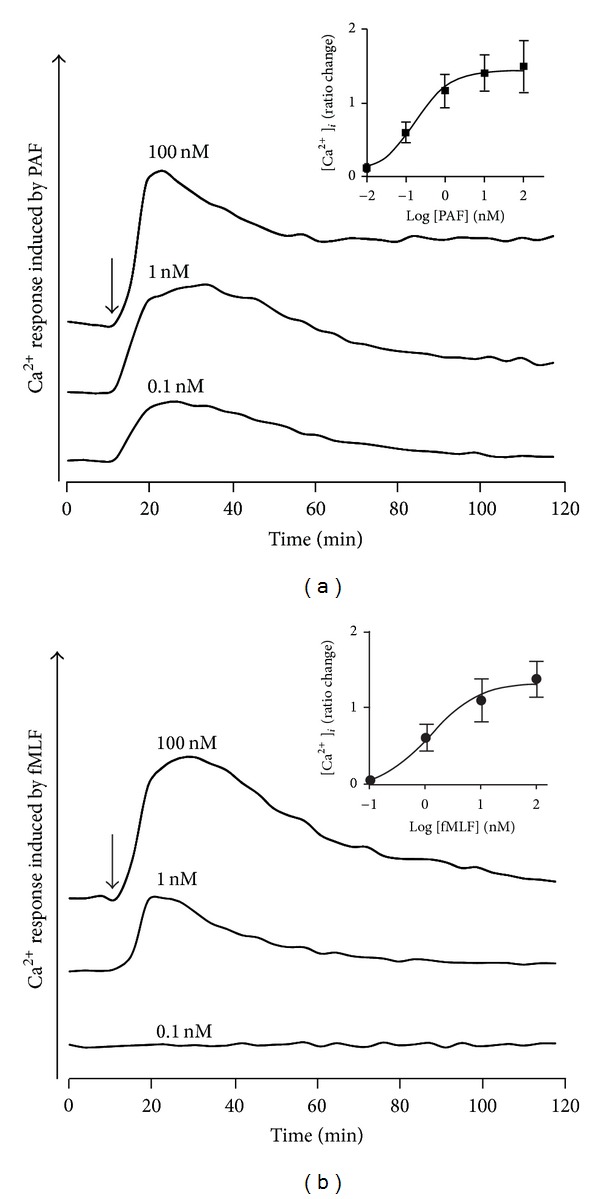
PAF induces a transient rise in intracellular Ca^2+^ in neutrophils. Fura-2 loaded neutrophils (2 × 10^6^/mL) were triggered with different concentrations of PAF (a) or fMLF (b), and the changes in fluorescence were followed using dual excitation at 340 nm and 380 nm, respectively, and an emission wavelength of 510 nm. A representative experiment out of at least three is shown, and an increase in Ca^2+^ is visualized as an increase in fluorescence when excited at 340 nm. To make a direct comparison easy, curves obtained with the different agonist concentrations are shown on top of each other. Abscissa, time of study (sec). The time point for addition of an agonist is marked by arrow. Inset: mean values of the transient rise in [Ca^2+^]_*i*_ (arbitrary units ± SEM (*n* = 3)).

Agonist binding to GPCRs initiates a chain of events, starting with dissociation of the receptor-associated G-protein and subsequently activation of a number of downstream signaling pathways [37, 38]. One such early pathway is the release of Ca^2+^ from intracellular stores and an increase in the cytosolic concentration of free calcium ions [Ca^2+^]_*i*_, resulting from the binding of the PIP_2_ cleavage product IP_3_ to its receptor located in storage organelles. Emptying of the storage organelles leads to the entry of extracellular Ca^2+^ through store-operated calcium channels in the plasma membrane, thereby prolonging the increase in [Ca^2+^]_*i*_. It has been suggested that some GPCRs, for example, FPR2, can directly open the channel in the plasma membrane without any involvement of the intracellular storage organelles, but we have earlier shown that signaling through FPR1 as well as through FPR2 involves primarily an emptying of the stores [39]. A rise in [Ca^2+^]_*i*_ depending on an emptying of the stores is by definition not inhibited through addition of a Ca^2+^ chelator (i.e., EGTA) ([40]; shown for fMLF in supplementary Figure 4). The PAF-induced increase in [Ca^2+^]_*i*_ was largely insensitive to EGTA suggesting that it primarily reflects a release of ions from intracellular stores (supplementary Figure 3). It should also be noticed that PAF is more potent than fMLF in inducing a rise in [Ca^2+^]_*i*_ with an EC_50_ value of 5 × 10^−10 ^M for PAF compared to 5 × 10^−9 ^M for fMLF (Figure 7). 

### 3.5. Distinct Utilization of Phosphoinositide 3-Kinase by fMLF and PAF

**Figure 8:**
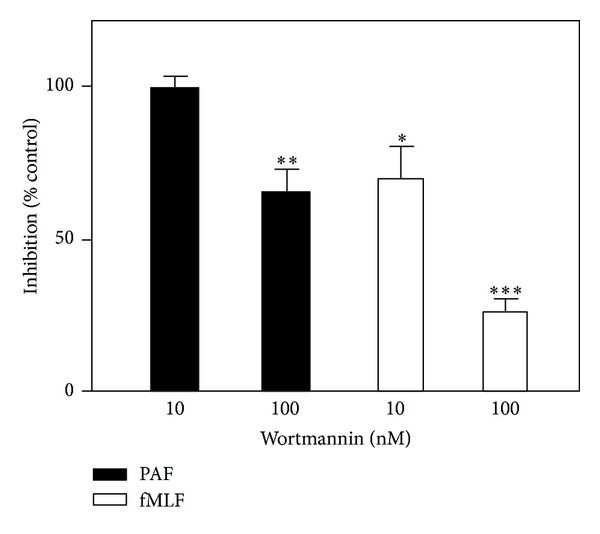
Effect of the PI3-kinase inhibitor wortmannin on the PAF and fMLF-induced release of superoxide anions. Human neutrophils (10^5^ cells) were incubated without (control), or with wortmannin (10 nM or 100 nM) for 10 min at 37°C. The cells were then stimulated with PAF (100 nM) or fMLF (100 nM), and the release of superoxidewas recorded continuously. Effects of wortmannin expressed in % of superoxide production (peak values compared) obtained without any additive (mean ± SEM; *n* = 3).

We have previously shown that both FPR1 (that binds fMLF) and CXCR2 (that binds IL-8) signal through the p38MAPK as well as the PI3 K pathways [21]. To determine whether there is a signaling difference between fMLF and PAF in activation the NADPH-oxidase, we used well-characterized pharmacological kinase inhibitors. The inhibitory effects of SB203580 (p38MAPK kinase inhibitor), Staurosporin, and RO318220 (protein kinase C inhibitors), and genistein (tyrosine kinase inhibitor) were the same for the fMLF and the PAF-induced responses (data not shown). The fMLF response was, however, found to be more sensitive than the PAF response to the PI3 K inhibitor wortmannin (Figure 8), suggesting that the PI3 K pathway is preferentially utilized by fMLF.

## 4. Discussion

Activation of neutrophils by the lipid chemoattractant PAF induces neutrophil responses typical of many other GPCRs agonists including a transient rise in intracellular calcium [Ca^2+^]_*i*_ accomplished by a mobilization of granule constituents to the cell surface, secretion of granule proteins, and ROS. Our interest to compare the similarities and differences between the PAFR and the FPRs arouses when we disclosed a fundamental difference in subcellular localization between the two receptors in human neutrophils. The two FPRs (FPR1 and FPR2) have earlier been shown to be localized to a minor part in the plasma membrane of resting cells, whereas the majority of the receptors are stored in mobilizable organelles, that is, the secretory vesicles the gelatinase granules, and the specific granules [18, 29]. In contrast, we show that the PAFR is localized primarily in the plasma membrane, and the functional differences between the neutrophil responses induced by PAF and the FPR1 agonist fMLF will be discussed in light of this fact. The difference in subcellular localization between the FPRs and the PAFR is most probably not directly related to the biophysical properties of the respective specific agonists, PAF being a lipid and the FPR agonists being peptides/proteins [4, 41]. This assumption is based on the fact that the receptors for the cytokine IL8 (CXCR1 and CXCR2) are also expressed solely in the plasma membrane [21], and this suggests that the precise localization of the receptors in resting neutrophils is instead related to their specific functions. Agonist/receptor pairs can be categorized as so-called end target chemoattractants/receptors or an intermediate group of attractants/receptors [1, 42]. In an inflammatory reaction, many chemoattractants are released from various locations including the vascular endothelium, the complement system, and possibly microbial intruders. Facing an environment with complex chemoattractants, neutrophils have to sense/respond and to make moving decision towards one or the other of multiple gradients of different chemoattractants. By definition, the group of intermediate attractants is generated at an early time point (possibly at the surface of endothelial cells) of the response, and these attractant also mediate their functions in the initial phase of the response, whereas the group of end target chemoattractants (possibly of microbial origin) is of importance for guiding the cells to the source of their generation in the tissue [21, 42]. It is reasonable to believe that the end-type chemoattractant receptors (FPR1, FPR2, and C5aR) needed to take action at the late stages of inflammatory process are primarily stored in the mobilizable organelles, whereas the intermediary ones (e.g., CXCR1/2, BLT1/2) involved in an early inflammatory response are readily expressed at the membrane surface. Whether PAFR should be categorized as “end” or “intermediary” type is not obvious as PAFR shares many similarities to CXCR1/2, but there are fundamental differences between the two receptors with respect to heterologous desensitization by FPR agonists. We have very recently demonstrated that the PAF response is not desensitized but actually primed by FPR1 agonists, and the primed PAF response involves a FPR-dependent signaling [43]. 

We have previously demonstrated a strong correlation between increased ROS production and surface receptor upregulation and suggested that receptor mobilization is a major mechanism for priming [17, 18, 20]. This suggestion was based on experiments/results with the FPR family of receptors which in resting neutrophils primarily are localized in mobilizable organelles. As mentioned, the receptors for IL-8 similar to PAFR are localized at the plasma membrane, and we have earlier presented results on IL-8 that support the suggested link between receptor localization/mobilization and priming [21, 44]. Our data showing that the PAF response terminates more rapidly than the fMLF response further support this link. It was, however, quite a surprise that the PAF-induced ROS response, in contrast to the IL-8 induced response, could be significantly primed by TNF*α* as well as by cytoskeleton disrupting drugs (this study). These data suggest that a novel mechanism is involved in priming of the PAF response. Alternative mechanisms to receptor mobilization have been proposed, and these include a translocation to the plasma membrane of signaling molecules such as protein kinase C, gp91^phox^, and p47^phox^/p67^phox^ (the membrane and cytosolic components, resp., of the NADPH-oxidase) or an elevation of intracellular calcium level per se [45–47]. The latter mechanism could, however, be excluded since no change in intracellular Ca^2+^ is induced by the priming agents used, and there is no direct link between oxidase activity and the cytosolic concentration of Ca^2+^ [48]. 

PAF has generally been considered as a poor ROS inducer and well recognized for its ability to attract neutrophil migration and for its function as a potent priming agent for enhanced production and release of superoxide anions upon exposure to a second stimulus [31, 33]. Using a technique with high sensitive and temporal resolution, to monitor ROS production in real time, we show that PAF directly activates neutrophils to produce superoxide (this study and [14]). The “intensity” of the PAF-induced response is somewhat lower than the fMLF induced response, and the duration is much shorter, characteristics that might explain the observations made by other investigators (using the much less sensitive cytochrome c reduction technique [49, 50]), that PAF is a very poor ROS inducer [13]. In our hands, PAF is indeed a potent ROS inducer in human neutrophils and the PAF response could be further amplified by priming agents. This knowledge should increase our understanding about the role of PAF in regulation of inflammatory responses.

We show that FPRs- but not PAFR-desensitized cells could be reactivated upon disruption of the actin cytoskeleton. It is clear that the PAF/PAFR induced response terminates much more rapidly than the fMLF/FPR1 response and that the mechanism for desensitization differ between the two receptors. A physical linkage/association to the actin cytoskeleton, of an agonist-occupied receptor, is an important mechanism for the termination of the superoxide anion production in neutrophils. It has been suggested that binding to the cytoskeleton of the ligand-receptor complex laterally separates the signaling receptor from the G-protein, a physical separation that terminates the signaling from the occupied receptor [34]. The fact that the fMLF as well as the PAF-induced responses were not only augmented but also prolongated in the presence Cyt B suggests that an association to the cytoskeleton constitutes an important mechanism for termination of both the FPR1 and the PAFR triggered responses. The responses are, however, terminated also in the presence of drugs that disrupt the cytoskeleton, suggesting that there are also other (nondefined) ways to terminate signaling. For FPR1, binding of the occupied receptors to the cytoskeleton is also an important mechanism for desensitization of receptors, and as a consequence, a disruption of the actin cytoskeleton reactivates the desensitized FPR1 [48]. We now show that desensitized PAFRs are not reactivated when the cytoskeleton is disrupted, meaning that the desensitization process is differently regulated for PAFR and FPR1. A functional uncoupling from G proteins and phosphorylation of PAFR, induced by the activated receptor itself, could be part of the termination process. Alternatively, rapid internalization and downregulation of the total number of PAFRs in neutrophils by PAF stimulation may form another part of the desensitization process [51, 52]. An immediate consequence of FPR1 and PAFR activation is the production of inositol 1,4,5-triphosphate and a subsequent transient elevation of intracellular Ca^2+^. The lipid remodeling is mediated by phosphoinositide kinases, phospholipase (PL) D, PLA_2_, and a phosphoinositide-specific PLC. The Ca^2+^ transient is, however, as mentioned, not linked to the generation of an NADPH-oxidase activating signal [48]. This notion is further supported by the fact that although PAF, is as potent as fMLF in inducing Ca^2+^ resposne, it is much weaker than fMLF in triggering NADPH-oxidase. The ligation of FPR1 and PAFR activates multiple signal cascades, and signaling through receptors for end target chemoattractants such as fMLF has been suggested to involve p38 MAPKs whereas the receptors recognizing inetermediary type chemoattractants such as IL-8 utilize the PI3 K/Akt pathway during neutrophil migration [42]. Determining the inhibitory profiles of p38 MAPK and PI3 K inhibitors on neutrophil oxidative response, we found that activation through end-type receptors as well as intermediate type receptors involve both p38 MAPK and PI3 K [21]. Stimulation with PAF as well as with fMLF result in equivalent phosphorylation and activation of p38 MAPK, however, a significant difference between FPR1/fMLF and PAFR/PAF; has been described with respect to p42/44 (ERK) MAPK activation [53]. In this study, we observed a signaling difference between FPR1 and PAFR with respect to the PI3 K pathway; that is, FPR1-mediated ROS production is more sensitive to the PI3 K inhibitor wortmannin than the PAF-mediated response. In summary, the data presented provide evidence that PAF can modulate neutrophil functions and directly promote the production of superoxide anion in addition to the many other known effects described for PAF, for example, priming, secretion, and receptor mobilization. These findings not only point out a possibility that PAF-mediated pathology may involve these yet unappreciated molecules released by a direct PAF stimulation, but they also strongly demonstrate that unique signaling pathways are utilized downstream of PAFR leading to priming effect and agonist driven desensitization. In addition, we show that there are fundamental differences between FPR1/FPR2 and PAFR with respect to their utilization of different mechanisms leading to oxidase activation/deactivation, and the difference between the receptors in their subcellular localization is possibly reflected in the signaling differences between FPR1/FPR2 and PAFR, similar to that earlier described between FPR1/FPR2 and CXCRs. The precise signaling pathways involved in priming and desensitization/reactivation of the PAFR as well as the missing direct link between signaling leading to a rise in cytosolic Ca^2+^, mobilization of granules, and activation of the oxidase in the fMLF/PAF response have to be further investigated. 

## Supplementary Material

Supplementaty Fig 1. Subcellular localization of the PAFR in resting neutrophils
Neutrophil subcellular organelles from disintegrated cells were fractionated on a two-layer Percoll gradient. Proteins from selected fractions were separated by SDS-PAGE and the localization of PAFR was determined by immunoblotting with a specific antibody against PAFR. The peak fractions for the azurophil granules (*α*), specific granules (*β*), and plasma membrame/secretory vesicles (*γ*), respectively, are shown by arrows.
Supplementary Fig 2. PAF and fMLF induce shedding of L-selectin and mobilization/secretion of neutrophil granule constituents
Human neutrophils (2 x 10^6^ cells) were activated by PAF or fMLF (various concentrations from 2 to 100 nM). The agonists were added to cells pre-warmed at 37°C and then incubated for 10 min. Control cells (C) were incubated at 37°C for the same time period but without any agonist added. The samples were centrifuged and the cell-free supernatants were used to determine release of granule constituents. The cell pellets were resuspended in KRG and used for the analysis of surface markers. Shedding of L-selectin (upper left) and CR3 mobilization (upper right) were examined by flow cytometry using specific fluorescence labeled antibodies against L-selectin and CR3, respectively. The results are given in percent of control (mean ± SEM; n=3). The amounts of NGAL (marker for the specific granules; lower left) and gelatinase (MMP9; marker for the gelatinase/specific granules; lower right) secreted from the cells were analyzed by ELISA and are expressed in ng/ml (mean± SEM; n=3) present in the cell free supernatants.
Supplementary Fig 3. Pertussis toxin (PTX) inhibits superoxide anion release induced by PAF and fMLF
Human neutrophils were incubated with PTX (500 ng/ml) at 37°C for various time periods as indicated. The cells were then activated with PAF (100 nM; black bars), fMLF (100 nM; white bars) or PMA (50 nM; grey bars) and the production of superoxide was determined as described. Neutrophils incubated at the same conditions but in the absence PTX (-PTX) were used as controls. The peak values of the responses were determined and the results are given as the respective ratio between +PTX treated and the control -PTX, and the data are from one respresentative experiment out of three. The incubation time with PTX is given in min and a ratio of 1 between +PTX/-PTX is expected when there is no effect of the pertusis toxin.
Supplementary Fig 4. A rise in intracellular Ca^2+^ is induced by PAF and fMLF also in extracellular Ca^2+^ is depleated prior to activation
Fura-2 loaded neutrophils (2 x 10^6^/ml) were triggered with fMLF (A) or PAF (B) and the change in fluorescence were followed. In order to remove extracellular Ca^2+^, EGTA at a final concentration of 2 mM (broken lines in A and B) was added 20 seconds before addition of an agonist. The rise in intracellular calcium was analyzed by monitoring Fura-2 fluorescence using dual excitation at 340 nm and 380 nm, and an emission wavelength of 510 nm. A representative experiment out of at least three is shown. Abscissa, time of study (sec); Ordinate, relative change in *[*Ca^2+^ *]*i.
