# Supplementary material for: The Subcellular Localization of the Receptor for Platelet-Activating Factor in Neutrophils Affects Signaling and Activation Characteristics
Source: Clin Dev Immunol. 2013 Aug 29;2013:456407. doi: 10.1155/2013/456407 (PMC3773398; doi:10.1155/2013/456407)

Supplementary Fig.1 Andréasson *et al*

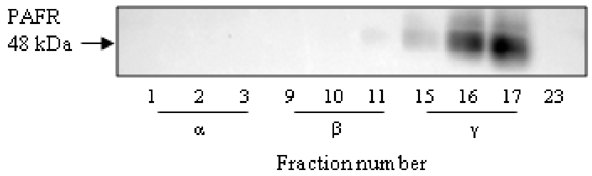

Supplementary Fig.2 Andréasson *et al*

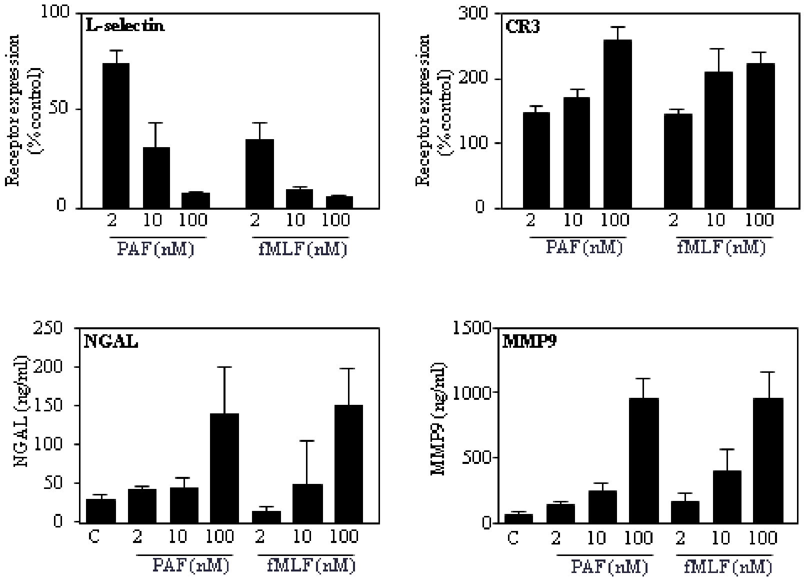

Supplementary Fig.3 Andréasson *et al*

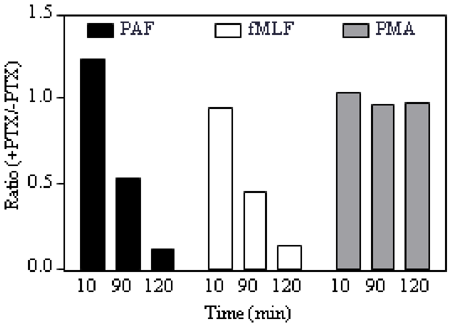

Supplementary Fig.4 Andréasson *et al*

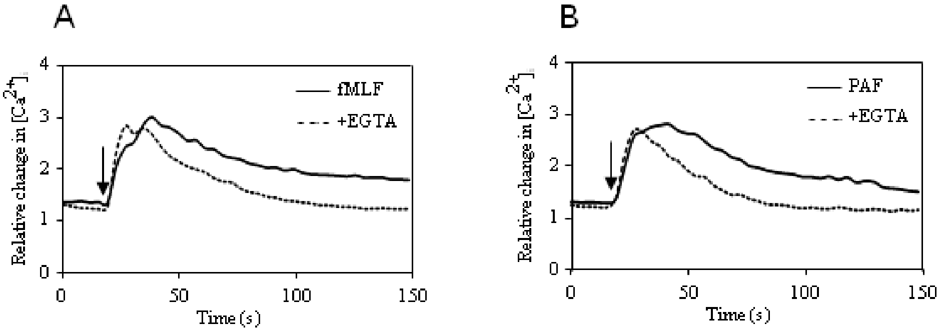

Supplement: Supplementary file 1 — Supplementaty Fig 1. Subcellular localization of the PAFR in resting neutrophils Neutrophil subcellular organelles from disintegrated cells were fractionated on a two-layer Percoll gradient. Proteins from selected fractions were separated by SDS-PAGE and the localization of PAFR was determined by immunoblotting with a specific antibody against PAFR. The peak fractions for the azurophil granules (α), specific granules (β), and plasma membrame/secretory vesicles (γ), respectively, are shown by arrows. Supplementary Fig 2. PAF and fMLF induce shedding of L-selectin and mobilization/secretion of neutrophil granule constituents Human neutrophils (2 x 10^6^ cells) were activated by PAF or fMLF (various concentrations from 2 to 100 nM). The agonists were added to cells pre-warmed at 37°C and then incubated for 10 min. Control cells (C) were incubated at 37°C for the same time period but without any agonist added. The samples were centrifuged and the cell-free supernatants were used to determine release of granule constituents. The cell pellets were resuspended in KRG and used for the analysis of surface markers. Shedding of L-selectin (upper left) and CR3 mobilization (upper right) were examined by flow cytometry using specific fluorescence labeled antibodies against L-selectin and CR3, respectively. The results are given in percent of control (mean ± SEM; n=3). The amounts of NGAL (marker for the specific granules; lower left) and gelatinase (MMP9; marker for the gelatinase/specific granules; lower right) secreted from the cells were analyzed by ELISA and are expressed in ng/ml (mean± SEM; n=3) present in the cell free supernatants. Supplementary Fig 3. Pertussis toxin (PTX) inhibits superoxide anion release induced by PAF and fMLF Human neutrophils were incubated with PTX (500 ng/ml) at 37°C for various time periods as indicated. The cells were then activated with PAF (100 nM; black bars), fMLF (100 nM; white bars) or PMA (50 nM; grey bars) and the production of superoxide [file 456407.f1.pdf]
